# Combined impact of sleep and obesity on female infertility in the NHANES 2017–2020

**DOI:** 10.1186/s12905-024-03164-2

**Published:** 2024-06-01

**Authors:** Zhe Wang, Yun-Hui Lai, Song-Yu Huang, Yu-Dong Liu, Shi-Ling Chen

**Affiliations:** Department of Obstetrics and Gynecology, Center for Reproductive Medicine, Nanfang Hospital, Southern Medical University, No 1838 Guangzhou Northern Road, Guangzhou, 510515 China

**Keywords:** Trouble sleeping/sleep duration, Overweight/obesity/ abdominal obesity, Female infertility, Combined effects

## Abstract

**Background:**

Sleep health and obesity may affect the risk of female infertility. However, few studies focused on the interaction of obesity and sleep health on the female infertility risk. This study aimed to evaluate the combined impact of trouble sleeping / sleep duration and overweight/obesity/ abdominal obesity on the risk of female infertility.

**Methods:**

The data for this cross-sectional study was obtained from National Health and Nutritional Examination Survey, which provided information on trouble sleeping, sleep duration, overweight/obesity, abdominal obesity, and confounding factors. Adopted weighted univariate and multivariate logistic regression models to explore the relationship between trouble sleeping, sleep duration, overweight/obesity, abdominal obesity, and the risk of infertility, respectively, and the combined effect of trouble sleeping and overweight/obesity, trouble sleeping and abdominal obesity, sleep duration and overweight/obesity, sleep duration and abdominal obesity, on the female infertility risk.

**Results:**

This study included a total of 1,577 women, and 191 were diagnosed with infertility. Women with infertility had a higher proportion of people with overweight/obesity, abdominal obesity, sleep duration ≤ 7 h and trouble sleeping than those with non-infertility. The result indicated that trouble sleeping [odds ratio (OR) = 2.25, 95% confidence intervals (CI): 1.49–3.39], sleep duration ≤ 7 h (OR = 1.59, 95% CI: 1.03–2.48), and the combined impact of abdominal obesity and trouble sleeping (OR = 2.18, 95% CI: 1.28–3.72), abdominal obesity and sleep duration ≤ 7 h (OR = 2.00, 95% CI: 1.17–3.40), overweight/obesity and trouble sleeping (OR = 2.29, 95% CI: 1.24–4.26), and overweight/obesity and sleep duration ≤ 7 h (OR = 1.88, 95% CI: 1.01–3.49) were associated with increased odds of infertility, respectively.

**Conclusion:**

There was combined effects of trouble sleeping/sleep duration ≤ 7 h and overweight/obesity/ abdominal obesity on increased odds of female infertility.

**Supplementary Information:**

The online version contains supplementary material available at 10.1186/s12905-024-03164-2.

## Background

Infertility is a common reproductive disease characterized by the failure to achieve a clinical pregnancy after 12 months of regular, unprotected sexual intercourse [1]. Globally, the prevalence of infertility among couples of reproductive age is estimated to be 15% [2]. Infertility not only affects women's reproductive health, but also has significant public health implications, including psychological disorders, societal repercussions, and marital discord [3, 4].

The importance of sleep health has gradually emerged as a growing concern within the realm of public health, given its pivotal role in metabolic and reproductive functions [5]. Previous studies have revealed that sleep parameters may affect the risk of infertility in women [6–8]. In the study of Zhao et al., reproductive-age women with sleep disorders exhibited a 2.14-fold increased risk of infertility compared to those without sleep disorders [8]. Short sleep duration and later sleep timing may constitute potential risk factors for female infertility [6, 7]. In addition, female obesity also might impair fertility through negative effects on ovulation control, oocyte, embryo, and endometrial development [9]. Research findings indicate that the risk of infertility in obese women was 2.7 times higher compared to that in normal-weight women [10]. It is worth noting that there exists a close association between obesity and sleep parameters. Both short and long sleep duration have been considered as risk factors for central obesity [11]. And obesity and sleep disorders may synergistically contribute to the potential pathological mechanisms of infertility, such as inflammation and insulin resistance [5, 12–14]. Therefore, when overweight/obesity and sleep problem appear at the same time, which might increase the risk of female infertility. However, to our knowledge, there were few studies on the direct relationship between the interaction of obesity and trouble sleeping on the female infertility risk.

The purpose of this study was to evaluate the interaction between trouble sleeping / sleep duration and overweight/obesity/ abdominal obesity on the risk of infertility based on the National Health and Nutritional Examination Survey (NHANES) database.

## Methods

### Study population

We selected the study population from NHANES database, a nationally representative, cross-sectional survey. The NHANES employs a multi-stage design that utilizes a stratified multistage probability sampling approach, and collects information of the health and nutrition in the U.S. population through interviews and physical examinations [15].

**Fig. 1:**
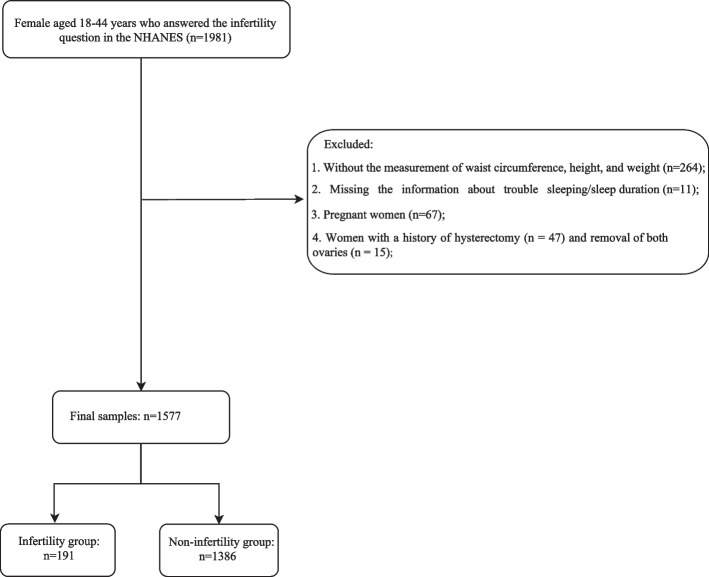
Flowchart of the selection process

The cross-sectional study utilized NHANES data from 2017–2020. A total of 1,981 female between the ages of 18 and 44 years who answered the infertility question were incorporated initially. After the exclusion of individuals without the measurement of waist circumference, height, and weight (*n* = 264), missing the information about trouble sleeping / sleep duration (*n* = 11), pregnant women (*n* = 67), women with a history of hysterectomy (*n* = 47) and removal of both ovaries (*n* = 15), 1,577 women were included in our final analysis (Fig. 1). The requirement of ethical approval for this was waived by the Institutional Review Board of Nanfang Hospital, Southern Medical University, because the data was accessed from NHANES (a publicly available database). The need for written informed consent was waived by the Institutional Review Board of Nanfang Hospital, Southern Medical University due to retrospective nature of the study. All methods were performed in accordance with the relevant guidelines and regulations.

### Outcome variable

The outcome variable of this study was infertility. The assessment of infertility was based on the responses to two questions from the NHANES reproductive health questionnaire [16]: (1) Have you ever attempted to become pregnant over a period of at least a year without becoming pregnant; and (2) Have you ever been to a doctor or medical provider because you have been unable to become pregnant? Any woman who answered affirmatively to either of these questions was considered infertility.

### Explanatory variables

The assessment of trouble sleeping is based on whether one meets any of the following question [17]: (1) The response to the question “Have you ever told a doctor or other health professional that you have trouble sleeping?” is affirmative; (2) the response is three or more nights per week when asked “How often do you snore, snort or stop breathing?”; (3) in response to the query “How often feel overly sleepy during day?”, the individual reported 16–30 times per month.

Sleep duration was assessed based on the response to the question [18] “How much sleep do you usually get at night on weekdays or workdays?” Participants were categorized into two groups: ≤ 7 h per night (insufficient sleep duration) and > 7 h per night (sufficient sleep duration).

The assessment of overweight/obesity was based on a body mass index (BMI) ≥ 25 kg/m^2^ [19]. Female abdominal obesity was defined as waist circumference ≥ 88 cm [20].

### Possible covariates

Possible covariates contained: age (years), race/ethnicity, education level, marital status, family poverty income ratio (PIR), smoke status, drink status, physical activity, age at menarche (years), work schedule, the number of deliveries live birth, menstrual cycle regularity, pelvic infection/pelvic inflammatory disease, hypertension, dyslipidemia, diabetes, hypersensitive C-reactive protein (hsCRP, mg/L), contraceptive pills, female hormones, and steroids.

Age at menarche was assessed by the question “When did you have your first menstruation?” Menstrual cycle regularity was assessed based on the response to the question “Do you currently have a regular menstrual cycle?” The assessment of pelvic inflammatory disease was conducted based on the reproductive health questionnaire from NHANES: "Have you ever received treatment for a pelvic infection/pelvic inflammatory disease?" Hypertension was diagnosed by systolic blood pressure ≥ 130 mm/Hg, or diastolic blood pressure ≥ 880 mm/Hg, or self-reported hypertension, or taking medicines of anti-hypertension drugs. Dyslipidemia was diagnosed by as total cholesterol ≥ 200 mg/dL or triglycerides ≥ 150 mg/dL, high-density lipoprotein ≤ 40 mg/dL, or low-density lipoprotein ≥ 130 mg/dL, or self-reported dyslipidemia, or utilization of cholesterol-lowering drugs. Diabetes was diagnosed by glycated hemoglobin A1c (HbA1c) levels ≥ 6.5%, fasting glucose levels ≥ 126 mg/dL, or 2 h oral glucose tolerance test ≥ 200 mg/dL, or self-reported diabetes, or utilization of diabetes medication or insulin. Work schedule was assessed using data from the Occupation Questionnaire Section, potential answers included (1) traditional 9 AM to 5 PM day, (2) evening or nights, (3) early mornings, (4) Variable (early mornings, days, and nights), and (5) unknown.

### Statistical analysis

Continuous variables were reported as mean (standard error, SE), and categorical variables were described as numbers and percentages. Independent sample t test was used to compare the two groups of continuous variables. Chi-squared test were used to compare the two groups of categorical variables. The missing variables were imputed using an imputation method, and sensitivity analysis was performed before and after interpolation (Supplementary Table 1).

A weighted univariate logistic regression analysis was employed to identify covariates associated with infertility (Supplementary Table 2). A significance level of *P* < 0.05 was used to determine statistical significance. Adopted weighted univariate and multivariate logistic regression models to explore the relationship between trouble sleeping, sleep duration, overweight/obesity, abdominal obesity, and the risk of infertility, respectively. Model I was unadjusted; Model II was adjusted for age, marital status, smoke status, the number of deliveries live birth, hypertension, and diabetes. Odds ratio (OR) was calculated with 95% confidence intervals (CI). Notably, we also constructed weighted univariate and multivariate logistic regression models to study the combined effect of trouble sleeping and overweight/obesity, trouble sleeping and abdominal obesity, sleep duration and overweight/obesity, sleep duration and abdominal obesity, on the infertility risk, respectively.

## Results

### Description of the study population

One thousand five hundred seventy-seven, female characteristics are shown in Table 1. The average age of women was 30.58 (SE, 0.26) years. Among them, 924 (57.10%) women exhibited abdominal obesity. 1035 (62.60%) women were classified as overweight/obesity. A total of 777 women experienced trouble sleeping. 350 women had insufficient sleep duration. What’s more, women with infertility were 191, and without infertility were 1,386. Women with infertility had a significantly higher age, smoking population ratio, proportion of people with hypertension, diabetes, overweight/obesity, abdominal obesity, sleep duration ≤ 7 h and trouble sleeping than those with non-infertility (*P* < 0.05).

**Table 1:** Characteristics of participants

Variables	Total (*n* = 1577)	Non-infertility group (*n* = 1386)	Infertility group (*n* = 191)	*P*
Age, years, Mean (S.E)	30.58 (0.26)	30.24 (0.25)	33.03 (0.59)	< 0.001
Race/ethnicity, n (%)				0.547
Mexican American	225 (11.83)	195 (11.64)	30 (13.21)	
Other Hispanic	162 (8.60)	139 (8.25)	23 (11.16)	
Non-Hispanic White	474 (54.92)	412 (54.96)	62 (54.65)	
Non-Hispanic Black	429 (13.77)	382 (13.83)	47 (13.34)	
Non-Hispanic Asian	190 (6.23)	168 (6.42)	22 (4.88)	
Other Race-Including Multi-Racial	97 (4.65)	90 (4.91)	7 (2.77)	
Education level, n (%)				0.396
Less than 9th grade/ 9-11th grade (Includes 12th grade with no diploma)	205 (8.98)	173 (8.56)	32 (12.02)	
High school graduate/GED or equivalent	323 (22.24)	283 (21.95)	40 (24.32)	
Some college or AA degree/ College graduate or above	1049 (68.78)	930 (69.49)	119 (63.66)	
Marital status, n (%)				< 0.001
Married/living with partner	872 (57.71)	735 (54.96)	137 (77.59)	
Widowed/divorced/separated	131 (6.87)	115 (7.00)	16 (5.95)	
Never married	574 (35.42)	536 (38.04)	38 (16.46)	
Family PIR, n (%)				0.226
< 1	409 (19.28)	372 (19.82)	37 (15.40)	
≥ 1	1168 (80.72)	1014 (80.18)	154 (84.60)	
Smoke status, n (%)				< 0.001
No	1156 (70.16)	1038 (72.20)	118 (55.39)	
Yes	421 (29.84)	348 (27.80)	73 (44.61)	
Drink status, n (%)				0.991
No	186 (7.48)	167 (7.48)	19 (7.50)	
Yes	1391 (92.52)	1219 (92.52)	172 (92.50)	
Physical activity, n (%)				0.110
< 750 MET· min/week	255 (14.73)	225 (14.94)	30 (13.18)	
≥ 750 MET· min/week	1015 (68.11)	905 (68.79)	110 (63.19)	
Unknown	307 (17.16)	256 (16.27)	51 (23.63)	
Age at menarche, years, n (%)				0.076
< 14	1229 (77.04)	1070 (76.25)	159 (82.73)	
≥ 14	348 (22.96)	316 (23.75)	32 (17.27)	
Menstrual cycle regularity, n (%)				0.265
No	117 (7.84)	102 (7.49)	15 (10.41)	
Yes	1460 (92.16)	1284 (92.51)	176 (89.59)	
Pelvic infection/ pelvic inflammatory disease, n (%)				0.196
No	1496 (95.65)	1320 (95.96)	176 (93.41)	
Yes	81 (4.35)	66 (4.04)	15 (6.59)	
Hypertension, n (%)				0.029
No	1376 (89.30)	1219 (90.17)	157 (83.02)	
Yes	201 (10.70)	167 (9.83)	34 (16.98)	
Dyslipidemia, n (%)				0.125
No	1018 (62.29)	915 (63.42)	103 (54.10)	
Yes	559 (37.71)	471 (36.58)	88 (45.90)	
Diabetes, n (%)				< 0.001
No	1480 (94.95)	1311 (95.78)	169 (89.01)	
Yes	97 (5.05)	75 (4.22)	22 (10.99)	
Central obesity, n (%)				0.001
No	653 (42.90)	594 (44.37)	59 (32.25)	
Yes	924 (57.10)	792 (55.63)	132 (67.75)	
Weight, n (%)				0.026
Underweight/normal weight	542 (37.40)	489 (38.61)	53 (28.65)	
Overweight/obesity	1035 (62.60)	897 (61.39)	138 (71.35)	
hsCRP, mg/L, Mean (S.E)	4.06 (0.21)	3.95 (0.21)	4.87 (0.64)	0.182
Contraceptive pills, n (%)				0.090
No	1368 (84.05)	1189 (83.02)	179 (91.53)	
Yes	209 (15.95)	197 (16.98)	12 (8.47)	
Female hormones, n (%)				0.078
No	1325 (80.07)	1155 (79.00)	170 (87.81)	
Yes	252 (19.93)	231 (21.00)	21 (12.19)	
Steroids, n (%)				0.246
No	1553 (98.05)	1366 (97.94)	187 (98.84)	
Yes	24 (1.95)	20 (2.06)	4 (1.16)	
The number of deliveries live birth, n (%)				< 0.001
0	6 (0.19)	3 (0.09)	3 (0.86)	
≥ 1	913 (54.59)	774 (51.59)	139 (76.29)	
Unknown	658 (45.22)	609 (48.31)	49 (22.86)	
Work schedule, n (%)				0.356
Traditional 9 AM to 5 PM day	434 (30.65)	382 (31.65)	52 (23.44)	
Evening or nights	156 (8.86)	131 (8.53)	25 (11.30)	
Early mornings	137 (7.55)	123 (7.77)	14 (5.94)	
Variable (early mornings, days, and nights)	378 (25.79)	336 (25.02)	42 (31.34)	
Unknown	472 (27.15)	414 (27.03)	58 (27.98)	
Sleep duration, n (%)				0.002
> 7 h	1227 (79.44)	1090 (80.89)	137 (68.93)	
≤ 7 h	350 (20.56)	296 (19.11)	54 (31.07)	
Trouble sleeping, n (%)				< 0.001
No	800 (50.91)	729 (53.70)	71 (30.72)	
Yes	777 (49.09)	657 (46.30)	120 (69.28)	

*GED* General educational development, *AA* Associate of arts, *PIR* Poverty income ratio, *MET* Metabolic equivalent, *hsCRP* hypersensitive C-reactive protein

### Relationship between trouble sleeping/sleep duration and infertility

As shown in Table 2, compared to individuals without trouble sleeping, those experiencing trouble sleeping were positively associated with the odds of infertility [(OR = 2.62, 95% CI: 1.79–3.82, *P* < 0.001) in Model I, and (OR = 2.25, 95% CI: 1.49–3.39, *P* < 0.001) in Model II]. Similarly, taking sleep duration of > 7 h as reference, sleep duration ≤ 7 h was related to an increased odds of infertility (Model I: OR = 1.91, 95% CI: 1.26–2.89, *P* = 0.004; Model II: OR = 1.59, 95% CI: 1.03–2.48, *P* = 0.039).

**Table 2:** Relationship between trouble sleeping, sleep duration, overweight/obesity, abdominal obesity, and infertility

Variables	Model I		Model II	
	**OR (95% CI)**	***P***	**OR (95% CI)**	***P***
Central obesity				
No	Ref		Ref	
Yes	1.68 (1.19–2.36)	0.005	1.25 (0.84–1.87)	0.254
Weight				
Underweight/normal weight	Ref		Ref	
Overweight/obesity	1.57 (1.01–2.43)	0.046	1.17 (0.72–1.92)	0.512
Sleep duration				
> 7 h	Ref		Ref	
≤ 7 h	1.91 (1.26–2.89)	0.004	1.59 (1.03–2.48)	0.039
Trouble sleeping				
No	Ref		Ref	
Yes	2.62 (1.79–3.82)	< 0.001	2.25 (1.49–3.39)	< 0.001

*OR* Odds ratio, *CI* Confidence interval

Model I: unadjusted

Model II adjusted for age, marital status, smoke status, the number of deliveries live birth, hypertension, and diabetes

### Relationship between overweight/obesity/ abdominal obesity and infertility

However, after adjusting for age, marital status, smoke status, the number of deliveries live birth, hypertension, and diabetes, the relationship between overweight/obesity/abdominal obesity and infertility did not reach statistical significance (Table 2, *P* > 0.05).

### Combined effect

After adjusting for covariates, the combined impact of abdominal obesity and trouble sleeping (OR = 2.18, 95% CI: 1.28–3.72, *P* = 0.006), abdominal obesity and sleep duration ≤ 7 h (OR = 2.00, 95% CI: 1.17–3.40, *P* = 0.013), overweight/obesity and trouble sleeping (OR = 2.29, 95% CI: 1.24–4.26, *P* = 0.011), and overweight/obesity and sleep duration ≤ 7 h (OR = 1.88, 95% CI: 1.01–3.49, *P* = 0.048) were found to be significantly associated with increased odds of infertility, respectively, as demonstrated in Table 3.

**Table 3:** Combined effect

Variables	Model I		Model II		
	**OR (95% CI)**	***P***	**OR (95% CI)**	***P***	***P* for Trend**
**Central obesity and Trouble sleeping**					
Central obesity = No and Trouble sleeping = No	Ref		Ref		< 0.001
Central obesity = Yes and Trouble sleeping = No	1.01 (0.55–1.84)	0.978	0.78 (0.41–1.49)	0.438	
Central obesity = No and Trouble sleeping = Yes	1.78 (0.70–4.53)	0.217	1.58 (0.63–3.95)	0.313	
Central obesity = Yes and Trouble sleeping = Yes	3.06 (1.90–4.92)	< 0.001	2.18 (1.28–3.72)	0.006	
**Central obesity and Sleep duration**					
Central obesity = No and Sleep duration > 7 h	Ref		Ref		0.012
Central obesity = Yes and Sleep duration > 7 h	1.75 (1.13–2.72)	0.014	1.32 (0.80–2.20)	0.268	
Central obesity = No and Sleep duration ≤ 7 h	2.14 (0.75–6.09)	0.147	1.78 (0.66–4.81)	0.242	
Central obesity = Yes and Sleep duration ≤ 7 h	3.12 (1.96–4.96)	< 0.001	2.00 (1.17–3.40)	0.013	
**Weight and Trouble sleeping**					
Underweight/normal weight and Trouble sleeping = No	Ref		Ref		0.001
Overweight/obesity and Trouble sleeping = No	1.31 (0.71–2.43)	0.369	1.03 (0.52–2.01)	0.934	
Underweight/normal weight and Trouble sleeping = Yes	2.52 (1.08–5.89)	0.034	2.25 (0.97–5.20)	0.057	
Overweight/obesity and Trouble sleeping = Yes	3.25 (1.89–5.61)	< 0.001	2.29 (1.24–4.26)	0.011	
**Weight and Sleep duration**					
Underweight/normal weight and Sleep duration > 7 h	Ref		Ref		0.022
Overweight/obesity and Sleep duration > 7 h	1.75 (1.01–3.03)	0.046	1.30 (0.72–2.38)	0.370	
Underweight/normal weight and Sleep duration ≤ 7 h	2.52 (0.83–7.66)	0.099	2.03 (0.69–5.95)	0.187	
Overweight/obesity and Sleep duration ≤ 7 h	2.92 (1.66–5.13)	< 0.001	1.88 (1.01–3.49)	0.048	

OR Odds ratio, CI Confidence interval

Model I: unadjusted

Model II adjusted for age, marital status, smoke status, the number of deliveries live birth, hypertension, and diabetes

## Discussion

In this study, we utilized NHANES data to assess the association of trouble sleeping/sleep duration and overweight/obesity/abdominal obesity with the odds of female infertility, and their combined impact on infertility. The findings indicated that trouble sleeping and sleep duration ≤ 7 h might be related to the odds of female infertility, and there was a combined impact between abdominal obesity and trouble sleeping, abdominal obesity and sleep duration ≤ 7 h, overweight/obesity and trouble sleeping, and overweight/obesity and sleep duration ≤ 7 h on infertility.

Our study identified trouble sleeping and sleep duration of ≤ 7 h as risk factors for the odds of female infertility. Sleep, as a primary lifestyle behavior, is regulated by human homeostasis and circadian rhythms. Additionally, reproductive hormones also exhibit close associations with circadian rhythms [21]. Consistent with previous research, reproductive health in women was found to be linked with trouble sleeping and inadequate sleep duration [22, 23]. Trouble sleeping and inadequate sleep duration may result in dysregulation of clock genes, thereby potentially leading to a decrease in ovarian reserve, altered sensitivity to reproductive hormones in oocytes, and diminished oocyte quality, ultimately impacting women's reproductive health [24–26]. Additionally, numerous studies have indicated that obesity in women may heighten the risk of chronic oligo-anovulation and infertility [27, 28]. This association could be attributed to the dysregulation of the hypothalamic-pituitary-ovarian axis, leading to ovulation dysfunction among obese individuals [28]. The present study, however, found no statistically significant association between overweight/obesity/abdominal obesity and infertility. This may be attributed to limitations in the different sources of sample size. In addition, considering that the NHANES database only collected information on infertility from 2017 to 2020, the data utilized in this study is derived exclusively from NHANES 2017–2020. However, it should be noted that NHANES 2017–2020 did not include records pertaining to insomnia, the duration of falling asleep and waking up during the night. Further research is warranted to delve deeper into the relationship between obesity and female infertility.

And more importantly, we found combined impacts between overweight/obesity abdominal obesity and trouble sleeping, and overweight/obesity abdominal obesity and sleep duration ≤ 7 h on infertility. To our knowledge, this is the first study assessing the combined impact trouble sleeping/sleep duration ≤ 7 h and overweight/obesity/abdominal obesity on the risk of female infertility. The possible mechanisms may explain the combined effects of trouble sleeping/sleep duration ≤ 7 h and overweight/obesity/ abdominal obesity on female infertility: (1) both obesity and poor sleep quality may activate the hypothalamic–pituitary–adrenal axis, which interfere the reproductive indices [23, 28]; (2) circadian dysrhythmia may result in infertility [29].

In a word, this study found that the combination of abdominal obesity and trouble sleeping, abdominal obesity and sleep duration ≤ 7 h, overweight/obesity and trouble sleeping, and overweight/obesity and sleep duration ≤ 7 h may increase the odds of female infertility. It has been suggested that women of reproductive age should actively pay attention to their body weight and sleep patterns, and coexisting trouble sleeping/insufficient sleep duration and overweight/obesity/ abdominal obesity may expose them to a higher risk of infertility. Nevertheless, the findings of this study should be interpreted with due to several limitations. First, the cross-sectional study design could not establish a causal relationship of trouble sleeping /sleep duration, overweight/obesity/abdominal obesity, and female infertility. Second, the measurement of trouble sleeping, sleep duration, and infertility was self-reported in this study, which were subjective rather than objective data and may be influenced by recall bias. Furthermore, self-report infertility may not be suitable for women seeking medical help who have been infertile for less than a year. Third, due to limitations of the NHANES database, we lack information regarding the duration of infertility, regularity of the sexual intercourse, quality of sperm, all the causes of infertility and the diagnosis of polycystic ovary syndrome (PCOS). Our sample was also limited to the U.S. population, and it is essential to validate our findings in diverse populations. Future research should use more clinical data of trouble sleeping /sleep duration and overweight/obesity/abdominal obesity to examine their effects on female infertility.

## Conclusion

Both trouble sleeping and sleep duration ≤ 7 h were associated with increased odds of female infertility. Moreover, there was combined effects of trouble sleeping/sleep duration ≤ 7 h and overweight/obesity/ abdominal obesity on increased odds of female infertility. The validation of our findings still requires further large-scale prospective studies.

### Supplementary Information

Supplementary Material 1.

## Data Availability

The datasets generated and/or analyzed during the current study are available in the NHANES database, https://wwwn.cdc.gov/nchs/nhanes/.

## Abbreviations

NHANES: National Health and Nutritional Examination Survey
BMI: Body mass index
PIR: Poverty income ratio
hsCRP: Hypersensitive C-reactive protein
SE: Standard error
OR: Odds ratio
CI: Confidence intervals

## References

[1] Vander Borght M, Wyns C (2018). Fertility and infertility: Definition and epidemiology. Clin Biochem.

[2] Infertility Workup for the Women's Health Specialist (2019). ACOG Committee Opinion, Number 781. Obstet Gynecol.

[3] Liang J, Chen X, Huang J, Nie W, Yang Q, Huang Q (2023). Implications of serum uric acid for female infertility: results from the national health and nutrition examination survey, 2013–2020. BMC Womens Health.

[4] Sun H, Gong TT, Jiang YT, Zhang S, Zhao YH, Wu QJ (2019). Global, regional, and national prevalence and disability-adjusted life-years for infertility in 195 countries and territories, 1990–2017: results from a global burden of disease study, 2017. Aging (Albany NY).

[5] Eisenberg E, Legro RS, Diamond MP, Huang H, O'Brien LM, Smith YR (2021). Sleep habits of women with infertility. J Clin Endocrinol Metab.

[6] Pimolsri C, Lyu X, Goldstein C, Fortin CN, Mumford SL, Smith YR (2021). Objective sleep duration and timing predicts completion of in vitro fertilization cycle. J Assist Reprod Genet.

[7] Liang Z, Liu J (2022). Sleep behavior and self-reported infertility: A cross-sectional analysis among U.S. Women. Front Endocrinol (Lausanne).

[8] Zhao J, Chen Q, Xue X (2023). Relationship between sleep disorders and female infertility among US reproductive-aged women. Sleep Breath..

[9] Marinelli S, Napoletano G, Straccamore M, Basile G (2022). Female obesity and infertility: outcomes and regulatory guidance. Acta Biomed.

[10] Jungheim ES, Travieso JL, Carson KR, Moley KH (2012). Obesity and reproductive function. Obstet Gynecol Clin North Am.

[11] Theorell-Haglöw J, Berglund L, Janson C, Lindberg E (2012). Sleep duration and central obesity in women - differences between short sleepers and long sleepers. Sleep Med.

[12] Vajravelu ME, Kindler JM, Zemel BS, Jawad A, Koren D, Brar P (2022). Visceral adiposity is related to insulin sensitivity and inflammation in adolescents with obesity and mild sleep disordered breathing. J Pediatr Endocrinol Metab.

[13] Lam JC, Mak JC, Ip MS (2012). Obesity, obstructive sleep apnoea and metabolic syndrome. Respirology.

[14] Romero-Corral A, Caples SM, Lopez-Jimenez F, Somers VK (2010). Interactions between obesity and obstructive sleep apnea: implications for treatment. Chest.

[15] Lin J, Lin X, Qiu J, You X, Xu J (2023). Association between heavy metals exposure and infertility among American women aged 20–44 years: A cross-sectional analysis from 2013 to 2018 NHANES data. Front Public Health.

[16] Wang R, Feng Y, Chen J, Chen Y, Ma F (2022). Association between polyunsaturated fatty acid intake and infertility among American women aged 20–44 years. Front Public Health.

[17] Scinicariello F, Buser MC, Feroe AG, Attanasio R (2017). Antimony and sleep-related disorders: NHANES 2005–2008. Environ Res.

[18] Makarem N, Alcantara C, Musick S, Quesada O, Sears DD, Chen Z (2022). Multidimensional Sleep Health Is Associated with Cardiovascular Disease Prevalence and Cardiometabolic Health in US Adults. Int J Environ Res Public Health.

[19] An Y, Li JN, Wang Y, Tian W, Li N (2023). Association of overweight and obesity with vertebral fractures: a systematic review and meta-analysis. Minerva Endocrinol (Torino)..

[20] Liu B, Du Y, Wu Y, Snetselaar LG, Wallace RB, Bao W (2021). Trends in obesity and adiposity measures by race or ethnicity among adults in the United States 2011–18: population based study. BMJ.

[21] Miller BH, Olson SL, Levine JE, Turek FW, Horton TH, Takahashi JS (2006). Vasopressin regulation of the proestrous luteinizing hormone surge in wild-type and Clock mutant mice. Biol Reprod.

[22] Beroukhim G, Esencan E, Seifer DB (2022). Impact of sleep patterns upon female neuroendocrinology and reproductive outcomes: a comprehensive review. Reprod Biol Endocrinol.

[23] Kloss JD, Perlis ML, Zamzow JA, Culnan EJ, Gracia CR (2015). Sleep, sleep disturbance, and fertility in women. Sleep Med Rev.

[24] Yao Q-Y, Yuan X-Q, Liu C, Du Y-Y, Yao Y-C, Wu L-J (2022). Associations of sleep characteristics with outcomes of IVF/ICSI treatment: a prospective cohort study. Hum Reprod.

[25] Zheng Y, Liu C, Li Y, Jiang H, Yang P, Tang J (2019). Loss-of-function mutations with circadian rhythm regulator Per1/Per2 lead to premature ovarian insufficiency†. Biol Reprod.

[26] Koritala BSC, Porter KI, Arshad OA, Gajula RP, Mitchell HD, Arman T (2021). Night shift schedule causes circadian dysregulation of DNA repair genes and elevated DNA damage in humans. J Pineal Res.

[27] Talmor A, Dunphy B (2015). Female obesity and infertility. Best Pract Res Clin Obstet Gynaecol.

[28] Broughton DE, Moley KH (2017). Obesity and female infertility: potential mediators of obesity's impact. Fertil Steril.

[29] Sridhar GR, Sanjana NS (2016). Sleep, circadian dysrhythmia, obesity and diabetes. World J Diabetes.

