# Supplementary material for: Combined impact of sleep and obesity on female infertility in the NHANES 2017–2020
Source: BMC Womens Health. 2024 Jun 1;24:315. doi: 10.1186/s12905-024-03164-2 (PMC11143667; doi:10.1186/s12905-024-03164-2)
Supplement: Supplementary file 1 — Supplementary Material 1. [file 12905_2024_3164_MOESM1_ESM.docx]

**Supplemental Table 1 Sensitivity analysis of before and after interpolation**

| **Variables** | **Before interpolation** | **After interpolation** | ***P*** |
| --- | --- | --- | --- |
| Education level, n (%) |  |  | 0.090 |
| Less than 9th grade/ 9-11th grade (Includes 12^th^ grade with no diploma) | 185 (8.67) | 205 (8.98) |  |
| High school graduate/GED or equivalent | 293 (22.72) | 323 (22.24) |  |
| Some college or AA degree/ College graduate or above | 931 (68.61) | 1049 (68.78) |  |
| Marital status, n (%) |  |  | 0.646 |
| Married/living with partner | 775 (58.07) | 872 (57.71) |  |
| Widowed/divorced/separated | 121 (6.88) | 131 (6.87) |  |
| Never married | 513 (35.06) | 574 (35.42) |  |
| Family PIR, n (%) |  |  | 0.407 |
| <1 | 362 (18.87) | 409 (19.28) |  |
| ≥1 | 1016 (81.13) | 1168 (80.72) |  |
| Age at menarche, n (%) |  |  | 0.861 |
| <14 | 1224 (77.05) | 1229 (77.04) |  |
| ≥14 | 345 (22.95) | 348 (22.96) |  |
| Pelvic infection/ pelvic inflammatory disease, n (%) |  |  | 0.074 |
| No | 1490 (95.64) | 1496 (95.65) |  |
| Yes | 81 (4.36) | 81 (4.35) |  |
| hsCRP, mg/L, Mean (S.E) | 4.00 (0.21) | 4.06 (0.21) | 0.287 |

GED=general educational development; AA=associate of arts; PIR=poverty income ratio; hsCRP=hypersensitive C-reactive protein.

**Supplemental Table 2 Selection of covariates by weighted univariate logistic regression analysis**

| **Variables** | **OR (95%CI)** | ***P*** |
| --- | --- | --- |
| Age | 1.05 (1.03-1.07) | <0.001 |
| Race/ethnicity |  |  |
| Mexican American | Ref |  |
| Other Hispanic | 1.19 (0.65-2.20) | 0.561 |
| Non-Hispanic White | 0.88 (0.48-1.61) | 0.659 |
| Non-Hispanic Black | 0.85 (0.50-1.46) | 0.541 |
| Non-Hispanic Asian | 0.67 (0.38-1.18) | 0.157 |
| Other Race - Including Multi-Racial | 0.50 (0.16-1.57) | 0.221 |
| Education level |  |  |
| Less than 9th grade/ 9-11th grade (Includes 12th grade with no diploma) | Ref |  |
| High school graduate/GED or equivalent | 0.79 (0.38-1.64) | 0.511 |
| Some college or AA degree/ College graduate or above | 0.65 (0.37-1.15) | 0.132 |
| Marital status |  |  |
| Married/living with partner | Ref |  |
| Widowed/divorced/separated | 0.60 (0.33-1.10) | 0.094 |
| Never married | 0.31 (0.21-0.46) | <0.001 |
| Family PIR |  |  |
| <1 | Ref |  |
| ≥1 | 1.36 (0.81-2.29) | 0.239 |
| Smoke status |  |  |
| No | Ref |  |
| Yes | 2.09 (1.49-2.93) | <0.001 |
| Drink status |  |  |
| No | Ref |  |
| Yes | 1.00 (0.53-1.88) | 0.991 |
| Physical activity |  |  |
| ＜750 MET· min/week | Ref |  |
| ≥750 MET· min/week | 1.04 (0.65-1.67) | 0.861 |
| Unknown | 1.65 (0.79-3.42) | 0.173 |
| Age at menarche |  |  |
| <14 | Ref |  |
| ≥14 | 0.67 (0.42-1.08) | 0.094 |
| Menstrual cycle regularity |  |  |
| No | Ref |  |
| Yes | 0.70 (0.35-1.38) | 0.286 |
| Pelvic infection/ pelvic inflammatory disease |  |  |
| No | Ref |  |
| Yes | 1.67 (0.73-3.84) | 0.213 |
| Hypertension |  |  |
| No | Ref |  |
| Yes | 1.88 (1.02-3.45) | 0.044 |
| Dyslipidemia |  |  |
| No | Ref |  |
| Yes | 1.47 (0.87-2.49) | 0.144 |
| Diabetes |  |  |
| No | Ref |  |
| Yes | 2.80 (1.51-5.18) | 0.002 |
| Contraceptive pills |  |  |
| No | Ref |  |
| Yes | 0.45 (0.17-1.22) | 0.113 |
| Female hormones |  |  |
| No | Ref |  |
| Yes | 0.52 (0.24-1.13) | 0.096 |
| Steroids |  |  |
| No | Ref |  |
| Yes | 0.56 (0.19-1.64) | 0.274 |
| hsCRP | 1.02 (0.99-1.05) | 0.114 |
| The number of deliveries live birth, n (%) |  |  |
| 0 | Ref |  |
| ≥1 | 0.16 (0.03-0.91) | 0.040 |
| Unknown | 0.05 (0.01-0.31) | 0.002 |
| Work schedule, n (%) |  |  |
| Traditional 9 AM to 5 PM day | Ref |  |
| Evening or nights | 1.79 (0.85-3.77) | 0.121 |
| Early mornings | 1.03 (0.38-2.79) | 0.948 |
| Variable (early mornings, days, and nights) | 1.69 (0.84-3.42) | 0.137 |
| Unknown | 1.40 (0.86-2.26) | 0.166 |

GED=general educational development; AA=associate of arts; PIR=poverty income ratio; MET= metabolic equivalent; hsCRP=hypersensitive C-reactive protein; OR=odds ratio; CI=confidence interval.
